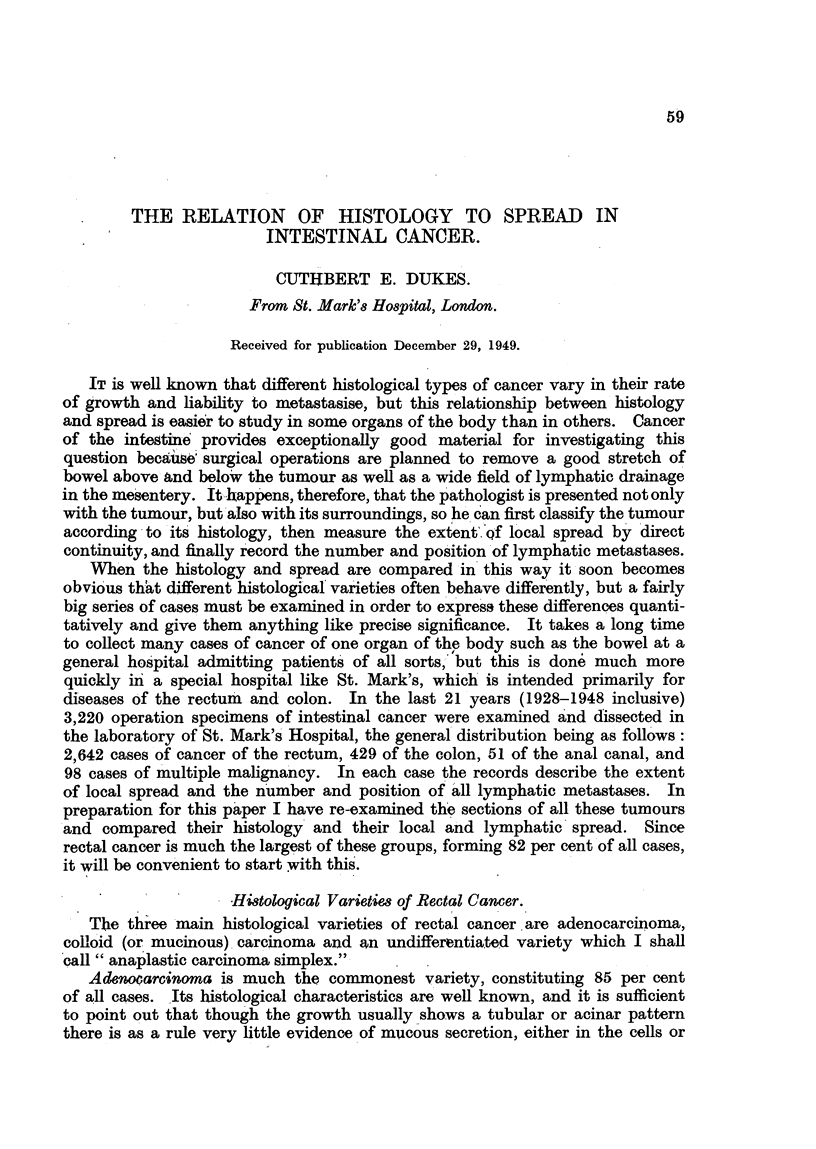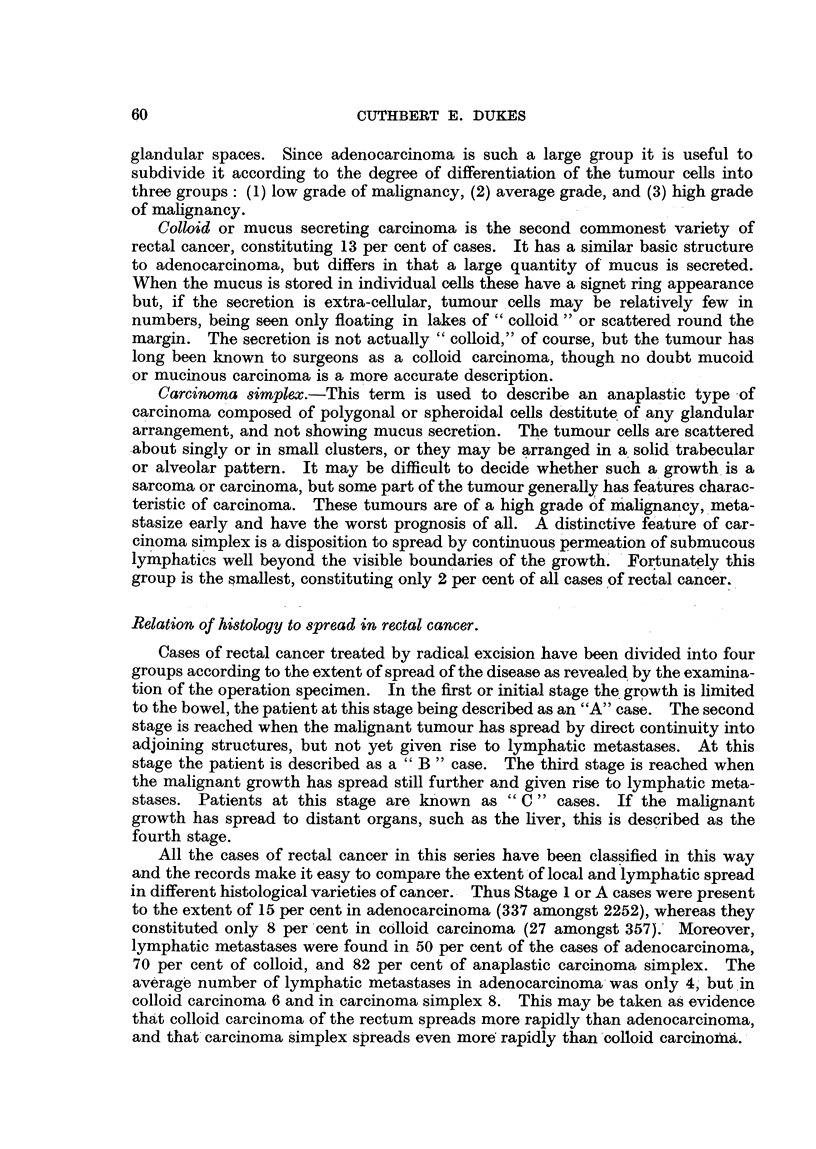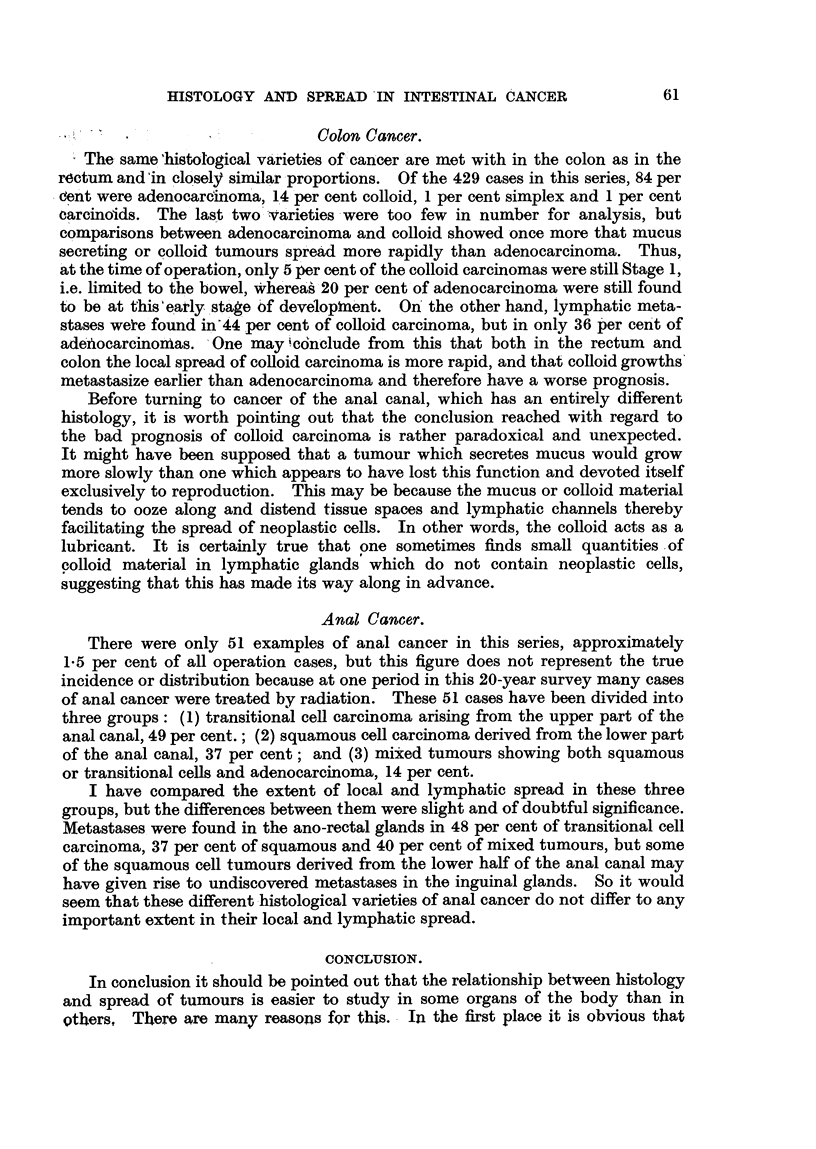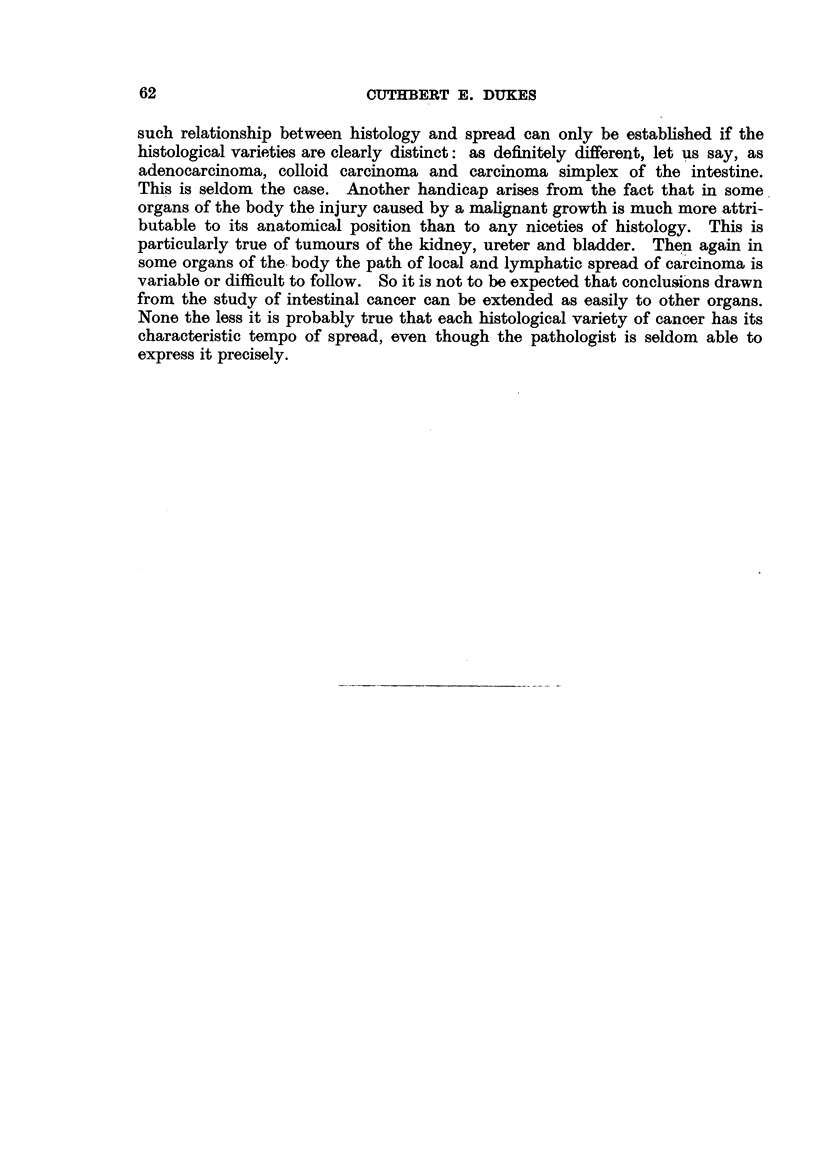# The Relation of Histology to Spread in Intestinal Cancer

**DOI:** 10.1038/bjc.1950.4

**Published:** 1950-03

**Authors:** Cuthbert E. Dukes


### THE RELATION OF HISTOLOGY TO SPREAD IN INTESTINAL CANCER.

CUTHBERT E. DUKES.

*From St. Mark's Hospital, London.*

Received for publication December 29, 1949.

It is well known that different histological types of cancer vary in their rate of growth and liability to metastasise, but this relationship between histology and spread is easier to study in some organs of the body than in others. Cancer of the intestine provides exceptionally good material for investigating this question because surgical operations are planned to remove a good stretch of bowel above and below the tumour as well as a wide field of lymphatic drainage in the mesentery. It happens, therefore, that the pathologist is presented not only with the tumour, but also with its surroundings, so he can first classify the tumour according to its histology, then measure the extent of local spread by direct continuity, and finally record the number and position of lymphatic metastases.

When the histology and spread are compared in this way it soon becomes obvious that different histological varieties often behave differently, but a fairly big series of cases must be examined in order to express these differences quantitatively and give them anything like precise significance. It takes a long time to collect many cases of cancer of one organ of the body such as the bowel at a general hospital admitting patients of all sorts, but this is done much more quickly in a special hospital like St. Mark's, which is intended primarily for diseases of the rectum and colon. In the last 21 years (1928–1948 inclusive) 3,220 operation specimens of intestinal cancer were examined and dissected in the laboratory of St. Mark's Hospital, the general distribution being as follows: 2,642 cases of cancer of the rectum, 429 of the colon, 51 of the anal canal, and 98 cases of multiple malignancy. In each case the records describe the extent of local spread and the number and position of all lymphatic metastases. In preparation for this paper I have re-examined the sections of all these tumours and compared their histology and their local and lymphatic spread. Since rectal cancer is much the largest of these groups, forming 82 per cent of all cases, it will be convenient to start with this.

### *Histological Varieties of Rectal Cancer.*

The three main histological varieties of rectal cancer are adenocarcinoma, colloid (or mucinous) carcinoma and an undifferentiated variety which I shall call "anaplastic carcinoma simplex."

*Adenocarcinoma* is much the commonest variety, constituting 85 per cent of all cases. Its histological characteristics are well known, and it is sufficient to point out that though the growth usually shows a tubular or acinar pattern there is as a rule very little evidence of mucous secretion, either in the cells or glandular spaces. Since adenocarcinoma is such a large group it is useful to subdivide it according to the degree of differentiation of the tumour cells into three groups : (1) low grade of malignancy, (2) average grade, and (3) high grade of malignancy.

*Colloid* or mucus secreting carcinoma is the second commonest variety of rectal cancer, constituting 13 per cent of cases. It has a similar basic structure to adenocarcinoma, but differs in that a large quantity of mucus is secreted. When the mucus is stored in individual cells these have a signet ring appearance but, if the secretion is extra-cellular, tumour cells may be relatively few in numbers, being seen only floating in lakes of " colloid " or scattered round the margin. The secretion is not actually " colloid," of course, but the tumour has long been known to surgeons as a colloid carcinoma, though no doubt mucoid or mucinous carcinoma is a more accurate description.

*Carcinoma simplex.*—This term is used to describe an anaplastic type of carcinoma composed of polygonal or spheroidal cells destitute of any glandular arrangement, and not showing mucus secretion. The tumour cells are scattered about singly or in small clusters, or they may be arranged in a solid trabecular or alveolar pattern. It may be difficult to decide whether such a growth is a sarcoma or carcinoma, but some part of the tumour generally has features characteristic of carcinoma. These tumours are of a high grade of malignancy, metastasize early and have the worst prognosis of all. A distinctive feature of carcinoma simplex is a disposition to spread by continuous permeation of submucous lymphatics well beyond the visible boundaries of the growth. Fortunately this group is the smallest, constituting only 2 per cent of all cases of rectal cancer.

### *Relation of histology to spread in rectal cancer.*

Cases of rectal cancer treated by radical excision have been divided into four groups according to the extent of spread of the disease as revealed by the examination of the operation specimen. In the first or initial stage the growth is limited to the bowel, the patient at this stage being described as an "A" case. The second stage is reached when the malignant tumour has spread by direct continuity into adjoining structures, but not yet given rise to lymphatic metastases. At this stage the patient is described as a " B " case. The third stage is reached when the malignant growth has spread still further and given rise to lymphatic metastases. Patients at this stage are known as " C " cases. If the malignant growth has spread to distant organs, such as the liver, this is described as the fourth stage.

All the cases of rectal cancer in this series have been classified in this way and the records make it easy to compare the extent of local and lymphatic spread in different histological varieties of cancer. Thus Stage 1 or A cases were present to the extent of 15 per cent in adenocarcinoma (337 amongst 2252), whereas they constituted only 8 per cent in colloid carcinoma (27 amongst 357). Moreover, lymphatic metastases were found in 50 per cent of the cases of adenocarcinoma, 70 per cent of colloid, and 82 per cent of anaplastic carcinoma simplex. The average number of lymphatic metastases in adenocarcinoma was only 4, but in colloid carcinoma 6 and in carcinoma simplex 8. This may be taken as evidence that colloid carcinoma of the rectum spreads more rapidly than adenocarcinoma, and that carcinoma simplex spreads even more rapidly than colloid carcinoma.

*Colon Cancer.*

The same histological varieties of cancer are met with in the colon as in the rectum and in closely similar proportions. Of the 429 cases in this series, 84 per cent were adenocarcinoma, 14 per cent colloid, 1 per cent simplex and 1 per cent carcinoids. The last two varieties were too few in number for analysis, but comparisons between adenocarcinoma and colloid showed once more that mucus secreting or colloid tumours spread more rapidly than adenocarcinoma. Thus, at the time of operation, only 5 per cent of the colloid carcinomas were still Stage 1, i.e. limited to the bowel, whereas 20 per cent of adenocarcinoma were still found to be at this early stage of development. On the other hand, lymphatic metastases were found in 44 per cent of colloid carcinoma, but in only 36 per cent of adenocarcinomas. One may conclude from this that both in the rectum and colon the local spread of colloid carcinoma is more rapid, and that colloid growths metastasize earlier than adenocarcinoma and therefore have a worse prognosis.

Before turning to cancer of the anal canal, which has an entirely different histology, it is worth pointing out that the conclusion reached with regard to the bad prognosis of colloid carcinoma is rather paradoxical and unexpected. It might have been supposed that a tumour which secretes mucus would grow more slowly than one which appears to have lost this function and devoted itself exclusively to reproduction. This may be because the mucus or colloid material tends to ooze along and distend tissue spaces and lymphatic channels thereby facilitating the spread of neoplastic cells. In other words, the colloid acts as a lubricant. It is certainly true that one sometimes finds small quantities of colloid material in lymphatic glands which do not contain neoplastic cells, suggesting that this has made its way along in advance.

*Anal Cancer.*

There were only 51 examples of anal cancer in this series, approximately 1·5 per cent of all operation cases, but this figure does not represent the true incidence or distribution because at one period in this 20-year survey many cases of anal cancer were treated by radiation. These 51 cases have been divided into three groups : (1) transitional cell carcinoma arising from the upper part of the anal canal, 49 per cent. ; (2) squamous cell carcinoma derived from the lower part of the anal canal, 37 per cent ; and (3) mixed tumours showing both squamous or transitional cells and adenocarcinoma, 14 per cent.

I have compared the extent of local and lymphatic spread in these three groups, but the differences between them were slight and of doubtful significance. Metastases were found in the ano-rectal glands in 48 per cent of transitional cell carcinoma, 37 per cent of squamous and 40 per cent of mixed tumours, but some of the squamous cell tumours derived from the lower half of the anal canal may have given rise to undiscovered metastases in the inguinal glands. So it would seem that these different histological varieties of anal cancer do not differ to any important extent in their local and lymphatic spread.

### CONCLUSION.

In conclusion it should be pointed out that the relationship between histology and spread of tumours is easier to study in some organs of the body than in others. There are many reasons for this. In the first place it is obvious that such relationship between histology and spread can only be established if the histological varieties are clearly distinct: as definitely different, let us say, as adenocarcinoma, colloid carcinoma and carcinoma simplex of the intestine. This is seldom the case. Another handicap arises from the fact that in some organs of the body the injury caused by a malignant growth is much more attributable to its anatomical position than to any niceties of histology. This is particularly true of tumours of the kidney, ureter and bladder. Then again in some organs of the body the path of local and lymphatic spread of carcinoma is variable or difficult to follow. So it is not to be expected that conclusions drawn from the study of intestinal cancer can be extended as easily to other organs. None the less it is probably true that each histological variety of cancer has its characteristic tempo of spread, even though the pathologist is seldom able to express it precisely.